# Characterization of Monomeric Intermediates during VSV Glycoprotein Structural Transition

**DOI:** 10.1371/journal.ppat.1002556

**Published:** 2012-02-23

**Authors:** Aurélie A. Albertini, Cécile Mérigoux, Sonia Libersou, Karine Madiona, Stéphane Bressanelli, Stéphane Roche, Jean Lepault, Ronald Melki, Patrice Vachette, Yves Gaudin

**Affiliations:** 1 Centre de Recherche de Gif, Laboratoire de Virologie Moléculaire et Structurale, CNRS (UPR 3296), Gif sur Yvette, France; 2 Institut de Biochimie et Biophysique Moléculaire et Cellulaire - Université de Paris-Sud 11, Orsay, France; 3 Centre de Recherche de Gif, Laboratoire d'Enzymologie et de Biochimie Structurale, CNRS (UPR 3082), Gif sur Yvette, France; The Salk Institute for Biological Studies, United States of America

## Abstract

Entry of enveloped viruses requires fusion of viral and cellular membranes, driven by conformational changes of viral glycoproteins. Crystal structures provide static pictures of pre- and post-fusion conformations of these proteins but the transition pathway remains elusive. Here, using several biophysical techniques, including analytical ultracentrifugation, circular dichroïsm, electron microscopy and small angle X-ray scattering, we have characterized the low-pH-induced fusogenic structural transition of a soluble form of vesicular stomatitis virus (VSV) glycoprotein G ectodomain (G_th_, aa residues 1–422, the fragment that was previously crystallized). While the post-fusion trimer is the major species detected at low pH, the pre-fusion trimer is not detected in solution. Rather, at high pH, G_th_ is a flexible monomer that explores a large conformational space. The monomeric population exhibits a marked pH-dependence and adopts more elongated conformations when pH decreases. Furthermore, large relative movements of domains are detected in absence of significant secondary structure modification. Solution studies are complemented by electron micrographs of negatively stained viral particles in which monomeric ectodomains of G are observed at the viral surface at both pH 7.5 and pH 6.7. We propose that the monomers are intermediates during the conformational change and thus that VSV G trimers dissociate at the viral surface during the structural transition.

## Author Summary

Vesicular stomatitis virus (VSV) enters cells via endocytosis. At low pH, its unique glycoprotein G catalyzes fusion between viral and endosomal membranes by going from a pre-fusion trimeric conformation to a post-fusion trimeric conformation. There is a lack of information about G structural intermediates during the transition, in particular topological issues concerning the transition pathway, as the structural rearrangement cannot occur without breaking the threefold symmetry. Using several biophysical techniques, we analyzed the structure of a soluble form of G ectodomain at several pH values to follow the structural transition. We identified flexible monomeric intermediates that explore a large conformational space. The monomeric population exhibits a marked pH-dependence and adopts more elongated conformations when the pH decreases. We propose that these monomers, also detected at the viral surface by electron microscopy, are intermediates during the fusion-associated conformational change and thus that VSV G ectodomain fully dissociates at the viral surface during the structural transition. This paper is an invitation to reconsider some data obtained on class I viral fusion glycoproteins (like Paramyxovirus F, influenza HA, HIV gp41) as for these proteins the topology of the conformational change is similar to that of VSV G.

## Introduction

Entry of enveloped viruses into host cells requires fusion of the viral envelope with the cellular membrane. This step is mediated by virally encoded glycoproteins, anchored in the viral membrane by a transmembrane (TM) domain, that undergo large structural rearrangements following interaction with specific triggers (e.g. a low pH environment and/or cellular receptors). These conformational changes result in the exposure of hydrophobic motifs (so-called “fusion peptides” or “fusion loops”), which then interact with one or both of the participating membranes, resulting in their destabilization and fusion. At the end of the refolding process, the fusion proteins are in a hairpin-like post-fusion structure, in which the fusion loop and TM domain are at the same end of the molecule and in the same fused membrane. Conformational change triggered in the absence of a target membrane inactivates the fusion properties of the fusogenic glycoprotein.

Determinations of the atomic structures of the ectodomains of many viral fusion glycoproteins in their pre- and/or post-fusion states have revealed a large diversity of conformations. Three different classes of viral fusion proteins have been identified to date based on their common structural motifs. Class I fusion proteins are characterized by their post-fusion structure: a trimer of hairpins containing a central alpha helical coiled-coil structure [1]–[3]. Class II fusion proteins are elongated molecules composed of beta structures that refold to form stable trimers of hairpin [4]–[7]. Class III fusion proteins combine structural elements found in the two other classes [8]–[11].

Three-dimensional structures provide static pictures of pre- and post-fusion conformations but the transition pathway still remains elusive. Nevertheless, all available data are consistent with the formation of an extended intermediate conformation [2]. In this putative conformation, the fusion peptides or fusion loops are exposed at the top of the molecule, distal from the viral membrane, and directed towards the target membrane. Class II fusion proteins are known to transit from a (homo- or hetero-) pre-fusion dimer to a post-fusion trimer through an intermediate monomer [12], [13]. Class I and class III fusion proteins are trimeric in both the pre- and post-fusion conformations [2], [9]. It is worth noting that for these proteins, the topology of the conformational change at the viral surface precludes going from the pre-fusion to the post-fusion counterpart without breaking the threefold symmetry, raising questions about the quaternary structure organization of the intermediates [14].

Vesicular stomatitis virus glycoprotein is the only class III fusion protein for which the structures of both the pre- and post-fusion states are available [11], [15]. Four distinct domains were identified in these two structures: a β-sheet-rich lateral domain, a central domain involved in trimerization, a pleckstrin homology domain (PH domain) and a fusion domain inserted into a loop of the PH domain. The fusion domain contains a membrane-interacting motif consisting of two hydrophobic loops located at the tip of an elongated three-stranded β-sheet.

The transition from the pre- to the post-fusion structure of VSV G involves a major reorganization of the molecule [10], [11], [15]. During this transition, the fusion domain is projected toward the target cell membrane through two structural changes: the reorganization of two hinge segments connecting the fusion domain to the PH domain, and lengthening of the central helix of the trimerization domain. Finally, the C-terminal domain associated with the transmembrane segment refolds into an α-helix that positions itself in the grooves of the trimeric core formed by the central helices in an antiparallel manner, to form a six-helix bundle. The post-fusion state thus has the typical “hairpin” structure with the fusion loops in the vicinity of the TM domains.

Besides these two crystal structures, that have been visualized at the viral surface [16], there is evidence for at least one other conformation, stabilized at lower temperature and intermediate pH, in which G is probably in an extended state allowing the fusion loops to interact with the target membrane [17]. All these different states of G are maintained in a pH-dependent equilibrium, which shifts towards the post-fusion state at low pH [18].

Furthermore, it has been shown that the pre-fusion trimer is unstable once solubilized by non-ionic detergent [19] and that there is an equilibrium between G protein trimers and monomers *in vivo* [20], [21]. This equilibrium probably also exists at the viral surface as suggested by previous reconstructions from EM tomography [16] showing that, at pH 7.5, only a few pre-fusion trimers could be detected.

Here, using several biophysical techniques, including analytical ultracentrifugation, circular dichroïsm (CD), electron microscopy and small angle X-ray scattering (SAXS), we have characterized the structure in solution of a thermolysin-generated VSV G ectodomain (G_th_, aa residues 1–422, the fragment that was previously crystallized) at several pH values. While the trimeric post-fusion conformation is the major species detected at low pH, the trimeric pre-fusion conformation is not detected in solution (even at high protein concentration). Rather, we demonstrate that at high pH, G is in a flexible monomeric state that explores a large conformational space. The equilibrium population of protein conformational states varies with pH: The conformational space explored by the protein at pH 7.5 appears to be shifted toward more elongated monomeric states than at pH 8.8. Furthermore, comparison between SAXS and CD data reveals that large relative movements of domains occur at pH values at which no significant modification of secondary structure content is detected. Solution studies are complemented by electron micrographs of negatively stained viral particles in which monomeric ectodomains of G are observed at the viral surface at both pH 7.5 and pH 6.7. We propose that the monomers are intermediates during the conformational change and thus that VSV G trimers dissociate at the viral surface during the structural transition.

## Results

### pH dependent interaction between G_th_ and liposomes

**Figure 1:**
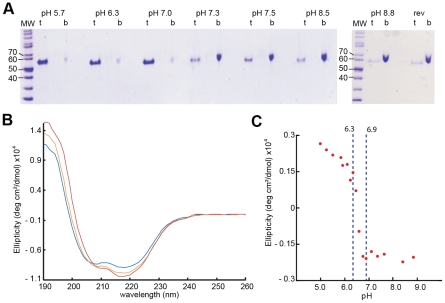
VSV G ectodomain structural transition. (A) Flotation of G_th_ with liposomes over a range of pH values from 5.7 to 8.8. For each experiment, top fraction (t) and bottom fraction (b) were collected and analyzed on 10% SDS PAGE stained with Coomassie Blue. The experiment “rev” shows the reversibility of the low-pH-induced association of G_th_ with liposomes after a re-incubation at pH 8.8. (B) Circular dichroïsm spectra of G_th_ at pH 8.8 (blue), pH 6.7 (orange) and pH 5.7 (red). Protein spectra were recorded at 15°C in 100 mM potassium phosphate buffer at a protein concentration of 0.8 mg/ml. (C) Plot of G_th_ ellipticity (measured at 200 nm) as a function of pH.

Interaction of VSV G with the target membrane occurs at an early stage of the fusion process [17]. We characterized this interaction by membrane flotation experiments. G_th_/liposome interaction was assessed over a broad pH-range, from pH 5.7 to pH 8.8 (Figure 1A). At pH 5.7, all the protein was located in the upper layer indicating a massive association of G_th_ with lipids. This massive binding to liposomes was detected up to pH 7.0. At pH 7.3 and above, most of the protein remained at the bottom of the tube indicating that, at these pH values, G_th_ poorly interacts with the membranes. Membrane association at low pH is reversible. Indeed, when a suspension of G_th_-lipid complexes generated at pH 5.7 was brought to pH 8.8, complete dissociation of proteins from liposomes was observed.

### G_th_ secondary structure transition

The pH-dependence of the secondary structure transition was analyzed by CD measurements carried out on G_th_ incubated at pH ranging from 5 to 8.8 (Figure 1B). The far-UV spectrum of G_th_ in both basic and acidic conditions exhibited two dichroïc bands at 208 and 220 nm indicating that the protein is structured in both extreme pH conditions.

Changes within G_th_ ellipticity at 200 nm (Figure 1C) as a function of pH revealed a single, sharp transition within the pH range 6.3–6.9. Such a curve can be interpreted in terms of a two-state equilibrium between two conformations predominantly populated at pH 7.0–8.8 and at pH 5–6.2 respectively. However, the absence of an isodichroïc point (Figure 1B) indicates that at least a third conformation exists that is significantly populated within the transition pH range.

CD spectra deconvolution allowed estimating the percent of canonical secondary structures present in the high and low pH conformations of G_th_ in solution. The estimated β-strand content (∼33%) was the same whether the protein was at pH 8.8 or pH 5.7. In contrast, helical structure proportion increased from 16% to 20% when decreasing pH. These values are in agreement with those found in respectively the pre- and post-fusion X-ray structures of G_th_ [11], [15].

We thus undertook the structural characterization of G_th_ in solution at pH 8.8 (pH of the crystal conditions for the pre-fusion structure), at pH 7.5 (physiological pH), at pH 6.7 (pH that corresponds to the mid-transition observed by CD) and at pH 5.7 (pH at which all spikes are in their post-fusion conformation at the viral surface).

### Oligomeric status of G_th_ as a function of pH

**Figure 2:**
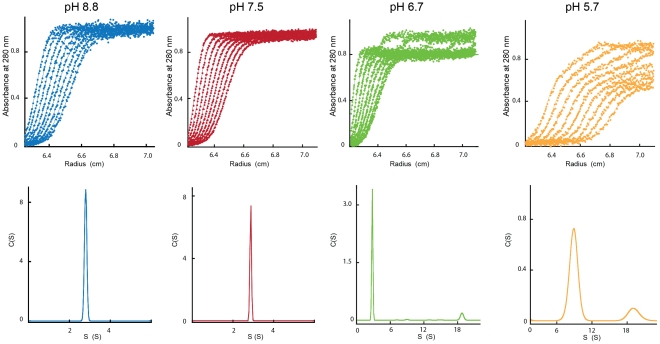
Analytical ultracentrifugation analysis of G_th_ at different pH values. The figure shows the experimental scans and data fitting curves (represented by a solid line) resulting from the analysis with Sedfit software (top panels) together with sedimentation coefficient distributions (bottom panels). Samples were spun at 45,000 rpm for G_th_ incubated at pH 8.8, pH 7.5 and pH 6.7 and at 20,000 rpm for G_th_ incubated at pH 5.7.

We investigated the oligomeric status of G_th_ in solution at various pH values by analytical ultracentrifugation. Sedimentation velocity analysis (Figure 2) was performed at two different protein concentrations (0.4 and 1.6 mg/ml).

At pH 8.8 and 7.5, at both protein concentrations, only one species of G_th_ was detected with a sedimentation velocity value of 2.8 S+/−0.14 S (*s20,w* = 3.4 S+/−0.17 S), too small for an oligomer and consistent with that expected for monomeric G_th_ [22].

At pH 5.7, two species were detected at both G_th_ concentrations with sedimentation coefficients *s_20,w_* = 10 S+/−0.42 S and 21 S+/−0.37 S. No monomeric species could be detected.

At pH 6.7, a monomeric species (*s_20,w_* = 3.4 S+/−0.13 S) was still detected together with a species with a sedimentation coefficient 21 S+/−0.33 S. A species with a sedimentation coefficient 10 S+/−0.6 S was also observed at higher G_th_ concentration. The proportions of the different species are given in Table 1.

**Table 1:** Relative abundance (in %) of the different G_th_ species derived from sedimentation velocity experiments performed at various pH values and G_th_ concentrations.

	pH 8.8	pH 7.5	pH 6.7			pH 5.7		
S _20,w_	3.4 S	3.4 S	3.4 S	10.0 S	21.0 S	3.4 S	10.0 S	21.0 S
Fractional concentration at 0.4 mg/ml (%)	100	100	87	0	13	0	71	29
Fractional concentration at 1.6 mg/ml (%)	100	100	36	40	24	0	55	45

We also performed equilibrium sedimentation analysis at pH 8.8 and 6.0 (Figure S1) at 0.4 mg/ml. As expected, at pH 8.8, the sedimentation profile revealed the presence of a single species with a molecular weight of 52 kDa *i.e.* a monomer. At pH 6.0, the sedimentation profile was fitted using non-interacting species model. The best fit to the data was obtained for a mixture of two species with molecular masses of about 320 and 1,600 kDa respectively (Figure S1). We conclude that the 10S species detected at low pH corresponds to two trimers of G_th_ most probably in their post-fusion conformation interacting through their fusion loops. Such an interaction is present inside both crystalline forms of the post-fusion conformation of G_th_ [11]. As for the 21 S species, we propose that it corresponds to rosette-like aggregates similar to those already described for other soluble ectodomains of viral fusion proteins in their post-fusion conformation [23]–[25].

### SAXS characterization of the structure of G_th_ in solution at pH 8.8 and 7.5

The homogeneity of our G_th_ samples at pH 8.8 and 7.5, as seen by analytical ultracentrifugation, prompted us to use small angle X-ray scattering (SAXS) to obtain information on the conformation of G_th_ in solution.

**Figure 3:**
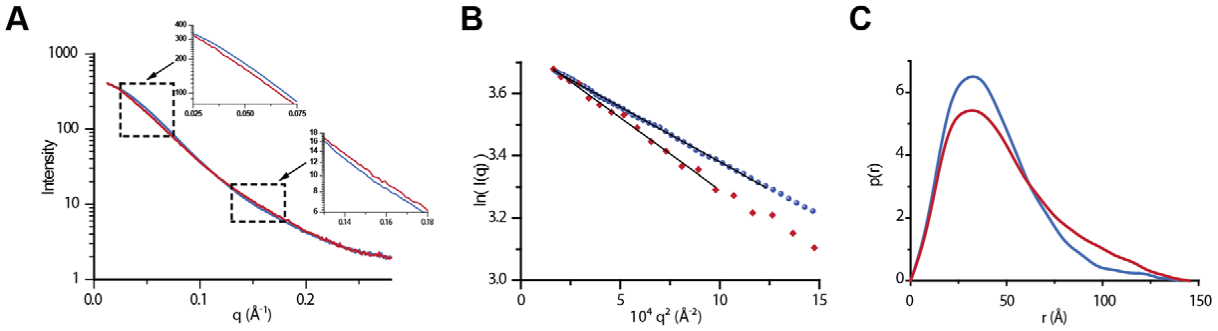
SAXS analysis of G_th_ in solution. (A) SAXS patterns of G_th_ at pH 8.8 (blue) and pH 7.5 (red). Scattering intensities are plotted against the momentum transfer q. The insets zoom on ranges of maximal differences between the two curves. Notice the color swap: blue curve above (resp. below) red one in the first (resp. second) inset. (B) Guinier plots calculated from the SAXS data shown in (A). (C) Distance distribution functions p(r) computed from SAXS experimental data at pH 8.8 (blue) and pH 7.5 (red).

The scattering patterns at pH 8.8 and 7.5 are shown in Figure 3A with their Guinier plots in Figure 3B and the distance distribution functions p(r) in Figure 3C. All structural parameters are presented in Table 2. At both pH values, the estimate of the molecular mass (53.5 kDa) is identical to the sequence derived value calculated for a monomer of G_th_ with associated sugar chains. Therefore, the protein appears to be monomeric in solution at pH 8.8 and 7.5 even at concentrations of a few mg/ml confirming and extending the observations made by analytical ultracentrifugation at lower protein concentration.

**Table 2:** Structural parameters derived from SAXS data.

pH	R_g_ (Å)	D_max_ (Å)	MM_exp_ (MM_seq_) (kDa)	χ pre-	χ post-	χ pre/post	χ pathway A	χ pathway B	χ pathways A & B
8.8	32.5±0.2	140±10	53.5 (53.63)	32.8	41.6	12.5	3.57	2.72	1.62
7.5	37.4±0.3	145±10	53.5 (53.63)	16	6.8	4.14	1.45	1.68	1.23
7.3	41.9±0.6	ND	52.5 (53.63)	ND	ND	ND	ND	ND	ND

R_g_, D_max_ and MM_exp_ are the radius of gyration, maximum size and molecular mass respectively calculated from SAXS data. MM_seq_ is the molecular mass of G_th_ calculated from the primary structure of the protein with two associated sugar chains.

χ pre-, χ post-, χ pre/post, χ pathway A, χ pathway B and χ pathways A & B denote the discrepancy between experimental data and scattering curves calculated from G_th_ pre- and post-fusion protomers, combination of both, and combinations using model conformations from trajectory A, trajectory B and a combination using all the conformations from pathways A and B. ND: not determined.

The two scattering curves, the Guinier plots and the p(r) profiles all exhibit significant differences (see insets in Figure 3A) indicating that the conformation of G_th_ at pH 8.8 is different from that at pH 7.5. The two Guinier plots yield radius of gyration (R_g_) values of 32.5 Å±0.2 Å and 37.4±0.3 Å at pH 8.8 and 7.5 respectively. The p(r) profiles, with a peak at small distances, around 30 Å, followed by a slow decrease towards large distances are suggestive of an elongated global conformation of the molecule at both pH values, irrespective of their differences.

Crystal structures of the trimeric protein in the pre- and post-fusion states have already been determined. The protomer of G_th_ adopts a very different shape in both structures, moderately elongated in the pre-fusion state and very extended in the post-fusion state. The scattering pattern of the protomer in each conformation was calculated using Crysol [26]. The two curves are shown in Figure S2 (pH 8.8 and pH 7.5) superimposed to the experimental data. Both exhibit major differences with respect to the experimental patterns, with very large χ-values (Table 2). To test whether in solution the monomeric conformations corresponding to the protomers of the two well-characterized trimeric states of the molecule could be in equilibrium, we tried to fit our data by a linear combination of the two patterns. Although improved, no satisfactory agreement could be obtained, the best fit corresponding to 55% pre- and 45% post-fusion patterns at pH 8.8 and to 25% pre- and 75% post-fusion patterns at pH 7.5 (Table 2 and Figure S2).

**Figure 4:**
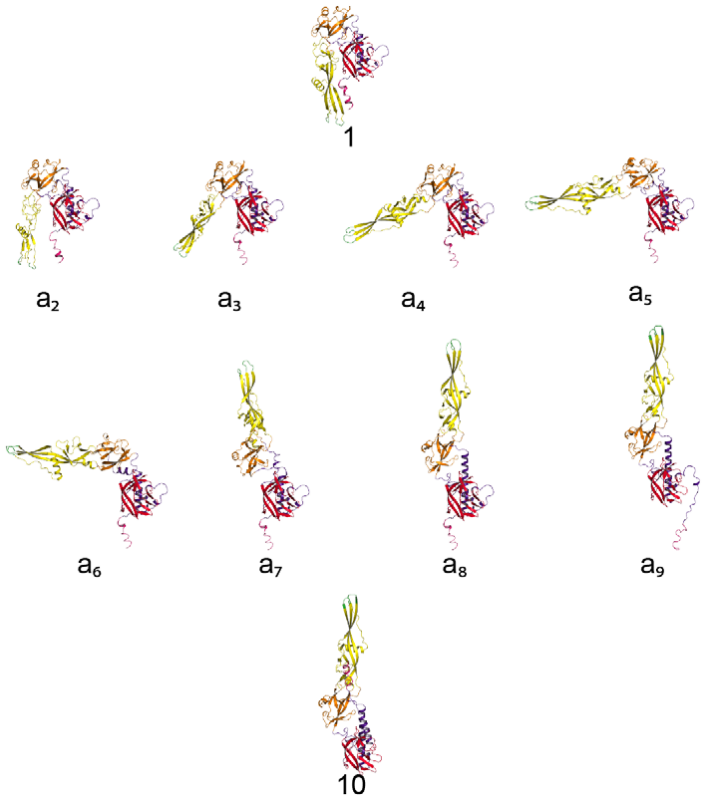
Models of structural intermediates during G_th_ structural transition. Conformation 1 is the G_th_ protomer found in the pre-fusion crystalline structure, conformation 10 is the G_th_ protomer found in the post-fusion crystalline structure. Conformations a2 to a9 were modeled using the Yale morph server (http://www.molmovdb.org/molmovdb/morph/). G_th_ molecule is colored by domains (lateral domain in red, oligomerization domain in blue, PH domain in orange and fusion domain in yellow) with the fusion loops in green and the C-terminus in pink.

**Figure 5:**
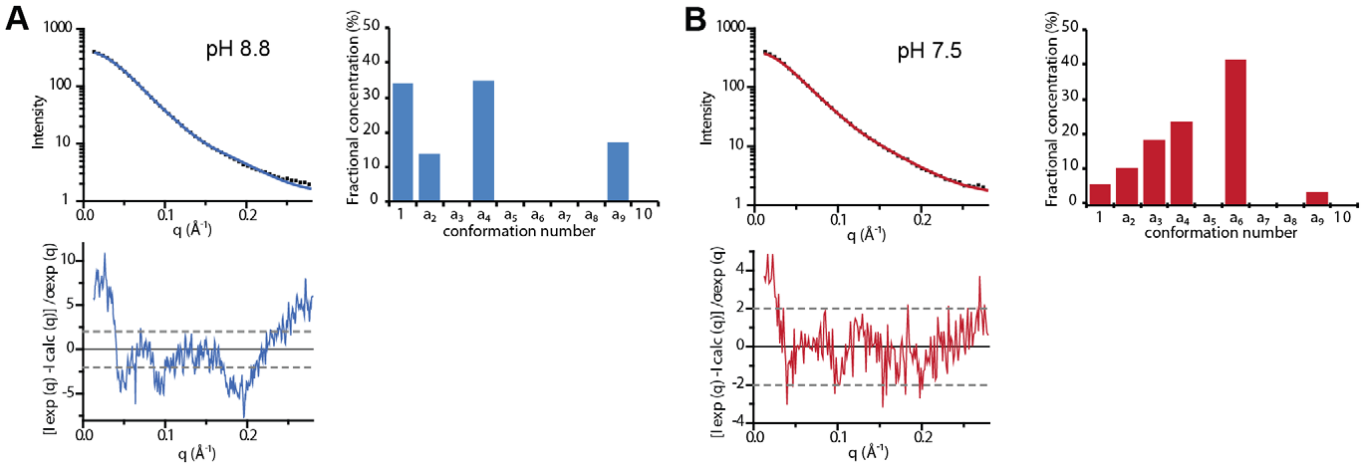
Fit of experimental SAXS patterns using linear combinations of calculated scattering curves from models shown in **Figure 4**. Panel A: pH 8.8; Panel B: pH 7.5. In each panel, top left frame: experimental scattering pattern (black dots) and the best fit from Oligomer (continuous blue or red line). Bottom left frame: distribution of reduced residuals ((I_exp_(q)−I_calc_(q))/σ_exp_(q)). Top right frame: histogram of the fractional concentrations of each conformation expressed in % of the total population. The distributions of reduced residuals between the best linear combination and the experimental curves although not flat and not restricted to the [−2, +2] band, notably in the smallest angle part, are much less structured than that of the two-structure fits (Figure S2).

The inability of the two known crystal structures, alone or in combination, to account for our solution data, indicates that at least a third, different conformation is present in solution under our experimental conditions (in agreement with CD data). We hypothesized that the molecule was very likely to explore a large conformational space rather than adopting a unique conformation. Indeed, although this is no proof of mobility, the scattering patterns appear to be essentially featureless, while the associated p(r) curves exhibit a trailing tail towards long distances as often observed in the case of a mixture of conformations [27]. An attempt was made at representing the main steps over a plausible path from the pre- to the post-fusion conformation, restricting the allowed changes to domains or large secondary structure elements (see Materials and Methods section for details). Eight putative intermediate conformations were thus modeled (conformations a2 to a9 in Figure 4). Their scattering patterns were calculated. That, together with the two crystal structure patterns, gave us an ensemble of 10 model curves (Figure 4). Using the program Oligomer [28], we determined the linear combination of these curves yielding the best fit to our experimental data. Although not ideal, the best fits represent a very significant improvement over the two-curve fits (compare Figure 5 and Figure S2, see also Table 2). The resulting distributions of fractional concentrations at pH 8.8 and 7.5 are shown to the right of the corresponding scattering patterns in Figure 5. They suggest that, while the majority of molecules are closer to the pre-fusion form or with a laterally displaced fusion domain at pH 8.8, a larger fraction of molecules adopt an elongated conformation at pH 7.5 at the expense of the pre-fusion like shapes (consistent with the increase of the apparent R_g_). We have selected here a trajectory from the pre- to the post-fusion conformation in which the fusion domain moves first. However, other trajectories can also be considered in which the C-terminal segment is displaced first with respect to the central domain followed by movements of the fusion domain. In a similar way to the first pathway, eight conformations were selected along this alternative trajectory (conformations b2 to b9 in Figure S3), their scattering patterns calculated and the same analysis performed using the program Oligomer (Figure S4). The corresponding χ-values and reduced residuals (Table 2 and Figure S4) suggest a comparable level of discrepancy, which does not make it possible to discriminate between the two proposed trajectories. We finally attempted to fit the scattering data leaving both pathways accessible to the protein. This yielded significantly improved fits for data recorded at both pH values (see Table 2 and Figure S4). Of note is the fact that whatever the pH considered and the set of conformations, at most 5 conformations are significantly populated. Furthermore, whatever the trajectory considered, the molecule appears to populate more elongated conformations when the pH is decreased from 8.8 to 7.5. This trend is confirmed by data recorded at pH 7.3 that give a still higher value of apparent R_g_ of 41.9 Å±0.6 Å with an estimate of 52.5 kDa for the molecular mass showing that the protein is still monomeric in solution. In this case, no fit using the ensemble of model curves is shown because of the limited quality of experimental data at q-values larger than 0.15 Å^−1^, but it can safely be concluded that G molecules at pH 7.3 adopt predominantly very elongated monomeric conformations.

### Characterization of G_th_ structure by electron microscopy at different pH values

**Figure 6:**
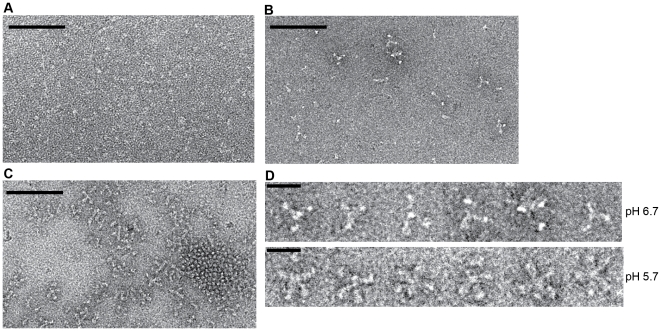
Electron microscopy on negatively stained G_th_ at pH 7.5, pH 6.7 and pH 5.7. (A) At pH 7.5, no oligomer of G_th_ is detected and a definite molecular shape cannot be identified. (B) At pH 6.7 a few G_th_ assemblies can be detected. (C) At pH 5.7 G_th_ trimers are observed assembled either in lattice via lateral interactions or in rosette-like assemblies. Protein concentration was 0.1 mg/mL for all 3 samples. The scale bar is for 100 nm. (D) Close up view of G_th_ rosettes observed at pH 6.7 and 5.7. Notice the differences in the aspect of the protein assemblies. The scale bar is for 20 nm.

We analyzed the various species previously identified by AUC and SAXS using electron microscopy and negative staining (Figure 6).

At pH 7.5 (Figure 6A), no aggregates were observed. No definite molecular shape could be identified due to G_th_ small size and/or flexibility.

At pH 5.7 (Figure 6C), only trimers with the characteristic shape of the post-fusion state were observed. They interact through their fusion loops to form rosettes made of a few trimers (about 6 to 10) that correspond to the 21S species. They also tend to cluster in closely packed bidimensional arrays in which trimeric heads of G_th_ were clearly seen (Figure 6). This network is probably the consequence of an interaction between G_th_ and the carbon film and is reminiscent of the closely packed arrays of G post-fusion form observed at the viral surface at low pH [16]. In the background, a few isolated dimers of trimers were also observed (Figure 6) that probably correspond to the 10S species observed by AUC.

At pH 6.7 (Figure 6B), no G_th_ trimers were observed. Although protein concentration was the same as at pH 5.7, much fewer protein particles were visible on the grid consistent with the ultracentrifugation data showing that most of the protein was monomeric in these conditions (and therefore not visible). G_th_ was only to be seen as part of rosette-like structures that appeared different from those observed at pH 5.7 (Figure 6D). Notably, G_th_ segments leading to the center of the assembly looked much thinner, suggesting that those G_th_ assemblies are made of G_th_ monomers interacting through their fusion domain.

### Visualization of monomeric G species at the viral surface

**Figure 7:**
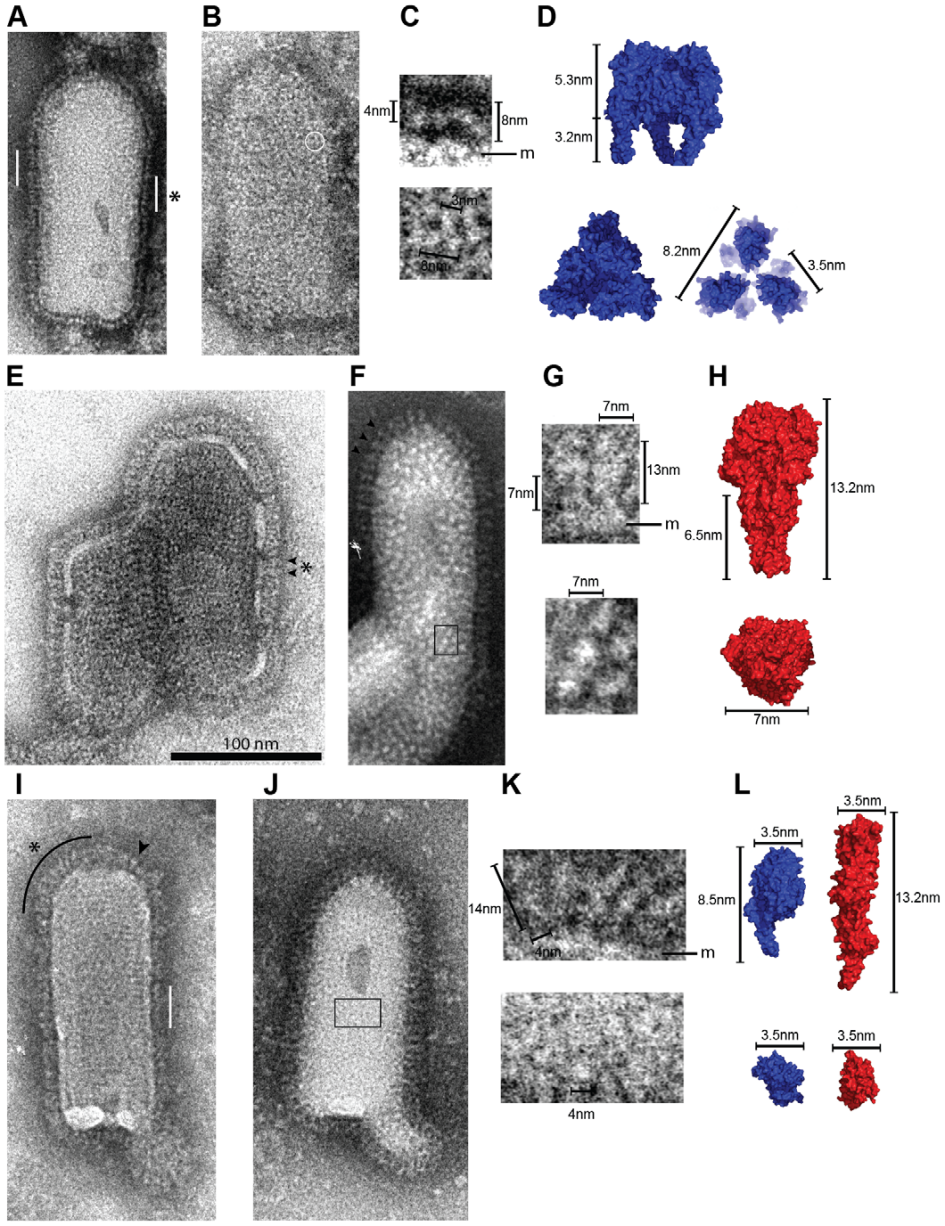
Morphology of G at the surface of negatively stained VSV particles at pH 7.5, 6.7 and 6.0. (A–B) VSV particles incubated at pH 7.5. G molecules form a thin and continuous layer of 8 nm height all around the viral particle. The white bars in A indicate areas where an even layer of G is visible. The circle on the viral particle in B shows a pre-fusion trimer of G in top view (Note that only a few trimers are visible). (C) Higher magnification of G layer region indicated by an asterisk in A (top) and of G pre-fusion trimer in top view located inside the circle in B (bottom). (D) Space filling model of VSV G X-ray trimer in its pre-fusion conformation as viewed from the side and from the top. The top view on the right is a clipped view of VSV G that illustrates what G pre-fusion trimers looks like at the viral surface. (E–F) VSV particles incubated at pH 6.0. Spikes are more elongated and can easily be seen individually. Arrowheads indicate some individual post-fusion trimers viewed from the side. Note that in panel F their regular spacing is particularly visible. Spikes can also be seen from the top in F, forming white domains having a diameter of about 7 nm. (G) Higher magnification of the region indicated by an asterisk on E showing spikes viewed from the side (top) and of the boxed region in F showing post-fusion trimers viewed from the top. (H) Space filling model of VSV G trimer in its post-fusion conformation as viewed from the side and from the top. (I–J) VSV particles briefly incubated at pH 6.7. G molecules form an irregular layer all around the viral particle. The white bar in I indicates an area with a layer of G that have kept their high-pH organization. The arrowhead in I points to a spike in its post-fusion conformation. The black line in I indicates an area where G forms a fuzzy heterogeneous layer in which some elongated rod like shape structures can be observed. Glycoproteins can also be seen from the top in J, forming small white domains having a diameter of about 4 nm. (K) Higher magnifications of G layer region indicated by an asterisk in I (top) and of the boxed region in J showing top views of G at the viral surface (bottom). (L) Space filling model of VSV G protomer in pre-fusion (in blue) and post-fusion (in red) conformations as viewed from the side and from the top. All VSV particles (A, B, E, F, I, J) are at the same scale (scale bar for 100 nm n in E). The images in C, G, and K are enlargements of these particles (magnification X4). Measurements and membrane location (m) are indicated.

We used negative staining to analyze the structure of VSV G at the viral surface. At pH 7.5, the virions appeared evenly distributed on the grid (Figure S5). We observed a rather continuous layer of spikes on the side of the viral particles in which individual glycoproteins were difficult to see (Figure 7A). In agreement with our previous data [16], the width of this layer (∼8 nm) and the width of the distal density (∼4 nm in white) (Figure 7C) were consistent with the length and the width of the head of the crystalline pre-fusion conformation (Figure 7D). When the stain allowed a visualization of the viral surface (Figure 7B), a homogeneous distribution of white domains of about 3 nm diameter (*i.e.* the size of a monomer head, Figure 7D) was observed. Although some of these domains seemed to form small equilateral triangle having the size of the pre-fusion conformation trimeric head (figure 7B and 7C), this was not the case for most of them, consistent with the presence of monomeric ectodomains.

At pH 6, as already described [16], viral particles were massively aggregated and many virions have fused together (Figure S5). When individual viral particles could be observed (Figure 7E and 7F), their spikes, when viewed from the side (Figure 7F and 7G), had the typical post-fusion conformation trimeric shape (Figure 7H) with their distal part thicker than the membrane proximal one. Furthermore, they were perpendicular to the membrane and regularly spaced (Figure 7F). When the viral surface was visible, a homogeneous distribution of white domains (with a diameter of about 7 nm) was observed. Therefore at this pH, most if not all the spikes were in the crystalline trimeric post-fusion conformation.

After short incubation at pH 6.7, viruses were slightly aggregated (Figure S5). At the surface of viral particles, the shape of the spikes was not homogeneous and several forms of G could be observed (Figure 7I). In some regions, G seemed to have kept its high pH organization as we could observe the characteristic 8 nm-wide layer (Figure 7I, white bar). Some spikes had also the typical post-fusion shape (Figure 7I, arrowhead). Besides these structures, glycoproteins appeared to form a fuzzy heterogeneous layer (Figure 7I and 7J, curved black line) in which some elongated rod like shape structures could be distinguished. The shape and dimensions of these glycoproteins, that were often oblique to the membrane, were consistent with those of an elongated monomer (Figure 7K top, and 7L). In top view (Figure 7J and 7K bottom), a homogeneous distribution of white domains of about 3 nm diameter, that did not seem to be organized neither to follow any symmetry, was observed, consistent here again with monomeric ectodomains.

## Discussion

Our structural characterization demonstrates that G_th_ is in a monomeric state in solution at high pH. We also provide evidences for the presence of monomers at the viral surface both at pH 7.5 and 6.7. At pH 7.5, in agreement with previous reconstructions from EM tomography [16], we show that only a few pre-fusion trimers can be detected and that most glycoproteins are likely in a monomeric state. At pH 6.7, rod-shaped elongated structures are also observed, the dimensions of which are consistent with a monomeric state.

We propose that in its monomeric state, G explores a manifold of conformations that are intermediates along the structural transition from the pre- to the post-fusion trimeric state. The following findings support this view.

First, both the pre- and post-fusion trimeric states can be generated and crystallized from the same high pH monomeric form. Indeed, the pre-fusion trimer is observed in the crystal [15], while the post-fusion trimer can be obtained by lowering the pH in solution (this study) and subsequently crystallized [11] in perfect agreement with what is expected from a *bona fide* intermediate.

Second, SAXS data establish that a significant fraction of monomers adopt a range of conformations that differ from that of the protomers in both the pre- and post-fusion trimeric structures.

Third, there are large differences between the pre- and post-fusion trimeric interfaces [15].

Fourth and last, as previously mentioned [10], [14], it is topologically impossible for pre-fusion trimeric G_th_ to reach the post-fusion state unless the threefold symmetry is lost, for example through dissociation into monomers.

**Figure 8:**
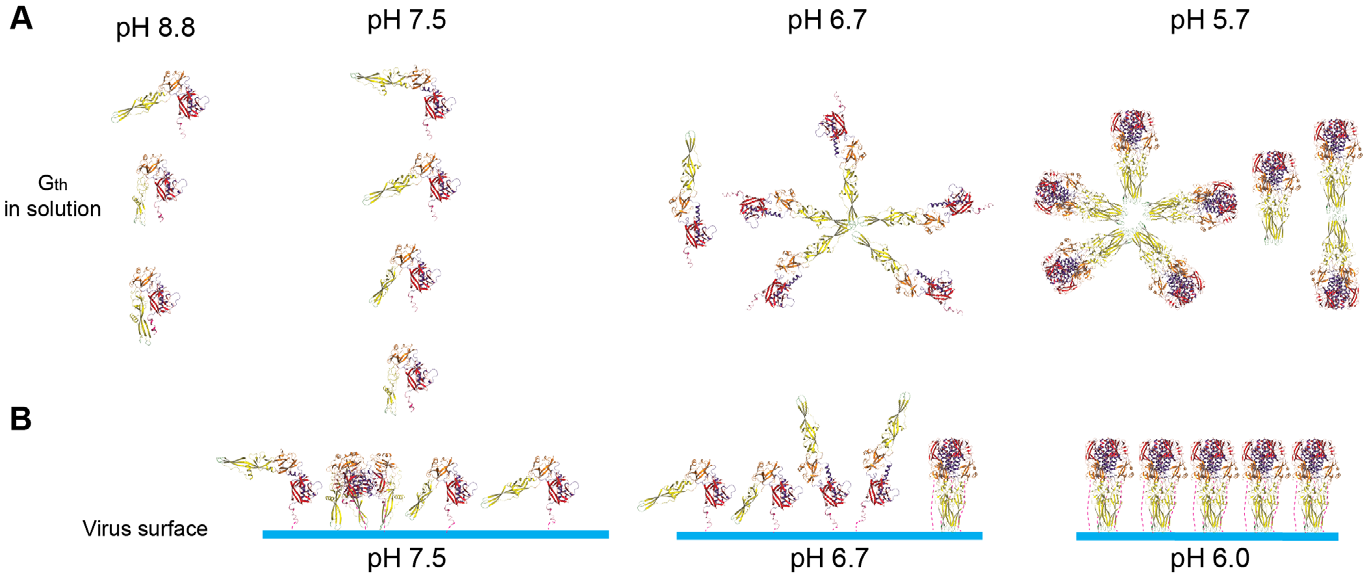
Pathway for the pH-dependent structural transition of G in solution and at the viral surface. (A) G_th_ species detected in solution at various pH. From pH 8.8 to pH 7.5 only monomers of G_th_ are present. pH decrease leads to monomers adopting in increasing number elongated shapes with the fusion loops exposed at the top of the protein. At pH 6.7, at low protein concentration, trimerization does not occur and G_th_ monomers associate to form rosettes through interaction via their fusion loops. Note that in this putative elongated conformation, C-terminal part (in magenta) and fusion loops are located at opposite extremities of the molecule and thus the C-terminal part cannot interfere with rosette formation through fusion loops aggregation. At still lower pH, monomers complete their refolding and reassociate to form post-fusion trimers. G_th_ post-fusion trimers have a strong tendency to interact through their fusion loops to form dimers of trimers and rosettes. (B) Plausible structural transition pathway of G at the viral surface. At pH 7.5, pre-fusion trimers and flexible monomers are in equilibrium at the viral surface. Lowering the pH to 6.7 favors the formation of elongated monomers oblique to the viral membrane with the fusion loops exposed at the top of the protein that favor an initial interaction of G with the target membrane. Some post-fusion trimers are already visible. At pH 6.0, all monomers have completed their refolding and reassociated to form post-fusion trimers that are regularly spaced at the viral surface. Color code for G_th_ is the same than in Figure 4.

The conformational space explored by monomeric G_th_ in solution exhibits a marked pH-dependence summarized in figure 8A. Indeed, the analysis of our SAXS data demonstrates that a higher fraction of G_th_ molecules adopts elongated conformations at pH 7.5 than at pH 8.8. Comparison with CD data reveals that these elongated states (significantly populated at pH 7.5) are detected in absence of significant change in the secondary structure content of G_th_. This indicates that large movements of domains can occur without elongation of the central helix nor formation of the lateral one that together make the 6-helix bundle structure found in the trimeric post-fusion conformation.

The flexible high-pH monomeric form is unable to bind target membranes and does not form rosettes in solution. In contrast, at intermediate pH (6.7), rosettes are observed that exhibit a different appearance from those formed by the post-fusion trimer at pH 5.7. They are visible at low protein concentration at which no trimer is detected, suggesting that they result from monomer association. This interpretation is consistent with the fact that G_th_ segments leading to the center of these aggregates are much thinner than those in the rosettes observed at pH 5.7. However, no side-chain is present at the tip of the fusion domain whose protonation state can change significantly within the pH range 8.8-6.7 [11]. Thus, the hydrophobicity of the loop region should remain unaltered over this pH range, which raises the question of the origin of rosette formation from monomers at pH 6.7. We propose that, at this pH, monomeric G_th_ explores preferentially very extended conformations that might resemble intermediates a6 to a9 (Figure 4) with the C-terminal part and fusion domain located at opposite extremities of the molecule. In such conformations the two hydrophobic fusion loops at the tip of the fusion domain reach out far into the solvent, and their unrestricted exposure makes mutual contact highly probable and energetically favorable, leading finally to rosette formation (Figure 8A, pH 6.7). This restricted conformational space would be favored by the protonation of histidine residues H60, H162 (located in the fusion domain part distal from the fusion loops) and H407 (located in the C-terminal part of the molecule) that are in close proximity in the pre-fusion conformation and have been proposed to play the role of pH-sensitive molecular switch [15].

Most probably, target membranes interact with the fusion loops whose access is essentially unhindered in proteins that adopt more elongated conformations. The presence of target membranes thereby shifts the equilibrium between the various forms toward the more elongated conformations and, as a consequence, affects the pH-dependence of the population landscape.

The major part of this work has been performed on a soluble form of G. One might argue that the absence of the transmembrane (TM) domain in G_th_ could affect the pathway of the structural transition and that the trimer of full-length G within the viral membrane could be stabilized through inter-TM domain interactions, by the increase in G local concentration or by optimization of G orientation for trimer interaction at the viral surface. However, when solubilized from the viral or cellular membranes at pH 7.4, full-length G has also been reported to be monomeric [19] and there is no evidence for any interaction between the TM domains of the three protomers within a pre-fusion trimer. In addition, our EM studies on viral particles show, in agreement with previous data [16], that at pH 7.5 most G molecules at the viral surface are not in the trimeric pre-fusion state and that at pH 6.7 monomeric elongated structures, oblique to the viral membrane, can also be observed. We may therefore confidently conclude that VSV G ectodomains can fully dissociate at the viral surface during the structural transition.

From these results, a plausible pathway for the structural transition is suggested in figure 8B. At high pH, G molecules at the viral surface are in equilibrium between the pre-fusion trimer and flexible monomers. Lowering the pH results in monomers adopting, in increasing number, elongated conformations with the fusion loops exposed at the top of the glycoprotein, thereby favoring the initial interaction with the target membrane. Finally, these monomers complete their refolding process to re-associate and form post-fusion trimers.

The topology of the structural transition of class I fusion glycoproteins is identical to that of VSV G [10]. For this class of fusion proteins, the initial steps leading to fusion peptide exposure and its interaction with the target membrane may maintain strict trimeric symmetry but the folding back of the C-terminal part of the molecule requires to break the three-fold symmetry of the molecule [14]. Remarkably, paramyxovirus F proteins (a representative of the class I fusion proteins) also exhibit large differences between their pre- and post-fusion trimeric interfaces [29], [30]. Thus, here again, a plausible scheme for the structural transition could also involve a manifold of transient short-lived monomeric intermediates. The scheme established here for a representative of class III fusion proteins could thus be a general feature of the structural transition of other viral fusion proteins.

## Materials and Methods

### Virus purification for G_th_ purification

Wild-type VSV (Mudd-Summer strain, Indiana serotype) was propagated in BSR cells, a clone of BHK 21. Around 20 hr *post-infection*, cell debris were first eliminated from the supernatant by filtration, then the virus was sent to the pellet by centrifugation at 4°C (3 hr 14,000 rpm in a JA14) and re-suspended in TD buffer (137 mM NaCl, 5 mM KCl, 0.7 mM Na_2_HPO_4_, 25 mM Tris-HCl pH 7.5). Viral purification was achieved by another centrifugation of 1 hr through a 25% glycerol cushion (in 10 mM Tris-HCl pH 7.5, 50 mM NaCl, 1 mM EDTA) at 25,000 rpm in a SW 28 rotor (Beckman) at 4°C. The pellet was re-suspended in TD buffer and the preparation was stored at −80°C for further use.

### Virus purification for EM

Wild-type VSV (Mudd-Summer strain, Indiana serotype) was propagated in BSR cells. Sixteen hours *post-infection*, cell debris were first eliminated from the supernatant centrifugation; then the virus was pelleted by centrifugation at 4°C (1.5 hr 14,000 rpm in a JA14) and gently re-suspended in TN buffer (150 mM NaCl, 10 mM Tris-HCl pH 7.5).

### G_th_ purification

Four volumes of concentrated virus (around 10 mg/ml) was thawed and incubated for 30 minutes at 37°C with one volume of phosphate-citrate buffer at pH 6.30 (prepared from a 0.1 M solution of citric acid and 0.2 M dibasic sodium phosphate stock solutions). Limited proteolysis with a 1 mg/ml solution of thermolysin was applied directly on viral particles (with a ratio viral solution/thermolysin solution of 10/1 v/v) during 1 hr at 37°C after which the digestion was stopped by addition of blocking buffer (900 mM Tris-HCl pH 8.8, 50 mM EDTA, supplemented with a cocktail of protease inhibitors). Then the soluble G ectodomain (G_th_) was separated from the “shaved” viral particle by centrifugation at 13,000 g during 45 minutes. This first supernatant was kept. The pellet was re-suspended in 50 mM Tris-HCl pH 8.8 and centrifuged for 30 minutes at 13,000 g and this second supernatant vas pooled with the first one. The pooled supernatant was loaded on the top of a 20% sucrose, 20 mM Tris-HCl pH 8.8 cushion and spun for 50 minutes at 40,000 rpm in a SW55 rotor. This supernatant was diluted 10 times in Tris-HCl 10 mM pH 8.8 and loaded on a DEAE trisacryl column (pre equilibrated with 20 mM Tris-HCl pH 8.8 buffer). G_th_ was eluted with 200 mM NaCl, 20 mM Tris-HCl pH 8.8 buffer. The eluted G_th_ was injected on a Superdex 200 HR10/30 column and eluted in 20 mM Tris-HCl pH 8.8 buffer. Fractions containing the G_th_ were pooled and concentrated with an amicon C/O 30 kDa ultrafiltration unit (Millipore).

### Liposome preparation for membrane flotation

Purified phosphatidylcholine (PC) from egg, phosphatidylethanolamine (PE) from egg and gangliosides from brain were purchased from Sigma and dissolved in a methanol∶chloroform mixture (ratio 1∶3). 700 µg of PC, 300 µg of PE and 100 µg of gangliosides were mixed in a round glass tube, the solvent was evaporated in Speed Vac yielding a thin lipid film on the sides of the tube. The lipid film was then hydrated in 150 mM NaCl to a final concentration of 5 mg/ml. Then the liposomes were bath sonicated during 20 minutes immediately before use. Liposomes were prepared fresh before each experiment.

### Liposome binding assay

100 µg of purified G_th_ at the desired pH was incubated with 500 µg of fresh liposomes during 30 minutes at 37°C (final volume 600 µL). The lipid-protein mix was mixed with a 80% w/v sucrose solution so that final concentration of sucrose was around 65%. The mixture was overlaid with 2 mL of 50% w/v sucrose solution w/v at the desired pH and 1.5 mL of 10% sucrose solution w/v at the desired pH. Cushions were spun 16 hr at 20°C in a sw55 Beckman rotor at 45,000 r.p.m. 20 µL of the top and bottom fraction was analyzed by SDS-PAGE. G_th_ in the top fraction was considered as liposome-bond, G_th_ in the bottom fraction was considered as not interacting with the membranes. For the reversibility experiment, lipid-protein mix was re-incubated at pH 8.8 after a first stage at pH 5.7.

### Circular dichroïsm

CD spectra were recorded on with a J810 (Jasco) spectropolarimeter using 0.02 cm path-length cuvette. G_th_ concentration was 0.8 mg/mL. All spectra were measured at 15°C in phosphate buffer and analyzed with the CDSSTR program of the software DichroWeb [31].

### Sedimentation velocity and equilibrium

Sedimentation velocity and equilibrium measurements were carried out using a Beckman Optima XL-A ultracentrifuge equipped with an AN60-Ti four-hole rotor and cells with two-channel 12 mm path length centerpieces. For sedimentation velocity measurements, 400 µl of protein were spun at 45000 rpm and at 15°C. Absorbance (at 280 nm and 290 nm for samples at 0.4 mg/ml and 1.6 mg/ml, respectively) displacement profiles were recorded every 5 min. Data were analyzed with the program Sedfit [32] and Svedberg [33]. For equilibrium experiments, 140 µl of protein were spun at 9000 rpm, 15°C, 26 hr, 10800 rpm, 24 hr, and 15600 rpm, 24 hr for samples at pH 8.8 and 4000 rpm, 48 hr, 4800 rpm, 29 hr, 7000 rpm, 20 hr for samples at pH 6.0. Absorbance was recorded at 295 nm and data were analyzed with the program Sedfit. The partial specific volume (0.7151 ml/g), buffers viscosities and densities at 20°C were calculated with the software Sednterp (pH 8.8 and pH 7.5: ρ = 1.00293 g/ml, η = 1.0169 cp; pH 6.7: ρ = 1.00963 g/ml, η = 1.0504 cp; pH 5.7: ρ = 1.00844 g/ml, η = 1.0456 cp).

### Small-angle X-ray scattering experiment and data processing

X-ray scattering data were collected at Synchrotron SOLEIL (Gif-sur-Yvette, France) on the beamline SWING (experiment at pH 8.8) and on a laboratory instrument (experiments at pH 7.5 and 7.3). G_th_ solutions were prepared in Tris-HCl buffer at the appropriate pH.

Data were analyzed using programs from the ATSAS package (Primus, Gnom, Crysol, Oligomer) [28] (see text S1 for details).

### Modeling

Plausible monomeric intermediates models with additional C-terminal missing segment (residues 414 to 422 for the pre-fusion state and residues 410 to 422 for the post-fusion state) and sugars were built with coot [34] using the PDB coordinates (2J6J and 2CMZ) (see text S1 for details).

### Electron microscopy

Purified G_th_ and virion were diluted in 150 mM NaCl in either 50 mM Tris-HCl pH 7.5, or 50 mM MOPS (3-(N-morpholino)propanesulfonic acid) at pH 6.7, pH 6.0 or pH 5.7. Samples (G_th_ and VSV particles) were then adsorbed onto airglow discharge carbon-coated grids and stained with sodium phospho-tungstic acid adjusted to the sample pH (7.5, 6.7, 6.0 and 5.7). Images were recorded in an electron microscope (model CM12; Philips) operated at 80 kV, with a nominal magnification of 35,000.

## Supporting Information

Figure S1 Sedimentation equilibrium of G_th_ at pH 8.8 and pH 6.0 at 0.4 mg/ml. Symbols represent experimental data and fitting curves are represented by a solid line. At pH 8.8 a single species with a molecular weight of 52 kDa was detected. At pH 6.0, data were fitted using a two non-interacting and non-exchangeable species model; the best fit to the data was obtained for a mixture of two species with molecular masses of about 320 kDa (30%) and 1,600 kDa (70%).
(TIF)

Figure S2 Calculated scattering intensity of pre- and post- fusion crystal structures of protomers and best fit of their linear combinations *vs* experimental data at pH 8.8 and pH 7.5. Top row: scattering curves; bottom row: reduced residuals corresponding to the linear combination fit, grey dashed lines indicate +2σ and −2σ. Color code: black dots: experimental data; blue line: calculated intensity of the protomer from the pre-fusion crystal structure; green line: calculated intensity of the protomer from the post-fusion crystal structure; red line: best fit using a linear combination of both pre- and post-fusion protomer calculated curves.
(TIF)

Figure S3 Second set of models of structural intermediates during G_th_ structural transition in which the C-terminal segment moves first (pathway B). Conformation 1 is the G_th_ protomer found in the pre-fusion crystalline structure, conformation 10 is the G_th_ protomer found in the post-fusion crystalline structure.
(TIF)

Figure S4 Comparison of fits of experimental data recorded at pH 8.8 and 7.5 using calculated scattering intensities of pathway A (10 models), pathway B (10 models), and combined pathways A and B (18 models). Top row: scattering curves; black dots: experimental data; continuous red line, best fit obtained using all 18 models of combined pathways. Middle row: distribution of reduced residuals corresponding to all three fits. blue: pathway A; yellow: pathway B; red: combined pathways; grey dashed lines indicate +2σ and −2σ. Bottom row: Histograms of the fractional concentrations of each conformation expressed in % of the total population for pathway B (yellow) and combined pathways A and B (blue).
(TIF)

Figure S5 Negatively stained VSV particles at pH 7.5, 6.7, 6.0 and 5.7. All images are at the same magnification (scale bar in the bottom right corner).
(TIF)

Text S1 Supporting information for SAXS experiment, data processing and modeling.
(DOC)
