# Supplementary figures and images for: Characterization of Monomeric Intermediates during VSV Glycoprotein Structural Transition
Source: PLoS Pathog. 2012 Feb 23;8(2):e1002556. doi: 10.1371/journal.ppat.1002556 (PMC3285605; doi:10.1371/journal.ppat.1002556)

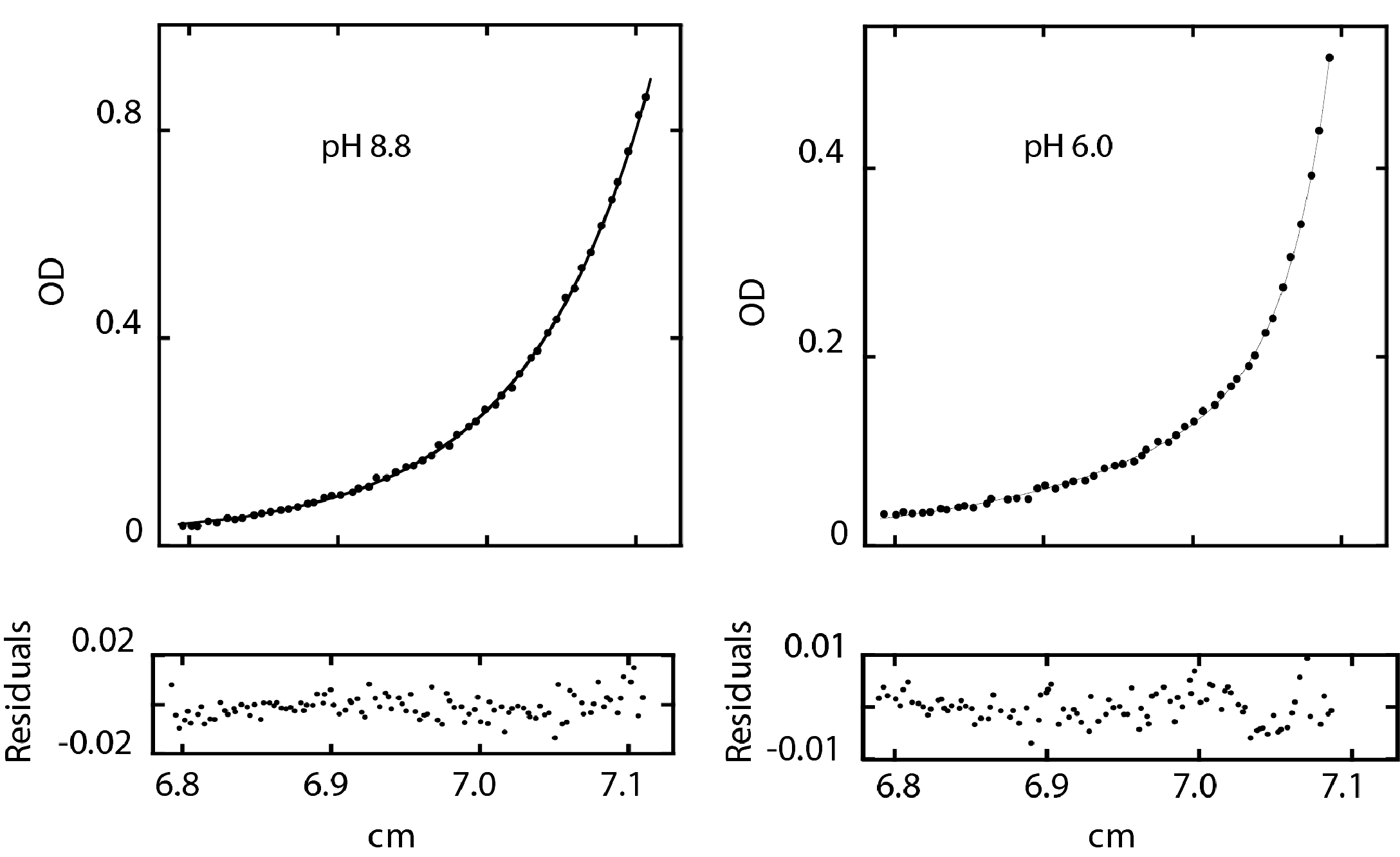

Supplement: Figure S1 — Sedimentation equilibrium of Gth at pH 8.8 and pH 6.0 at 0.4 mg/ml. Symbols represent experimental data and fitting curves are represented by a solid line. At pH 8.8 a single species with a molecular weight of 52 kDa was detected. At pH 6.0, data were fitted using a two non-interacting and non-exchangeable species model; the best fit to the data was obtained for a mixture of two species with molecular masses of about 320 kDa (30%) and 1,600 kDa (70%). (TIF) [file ppat.1002556.s001.tif]

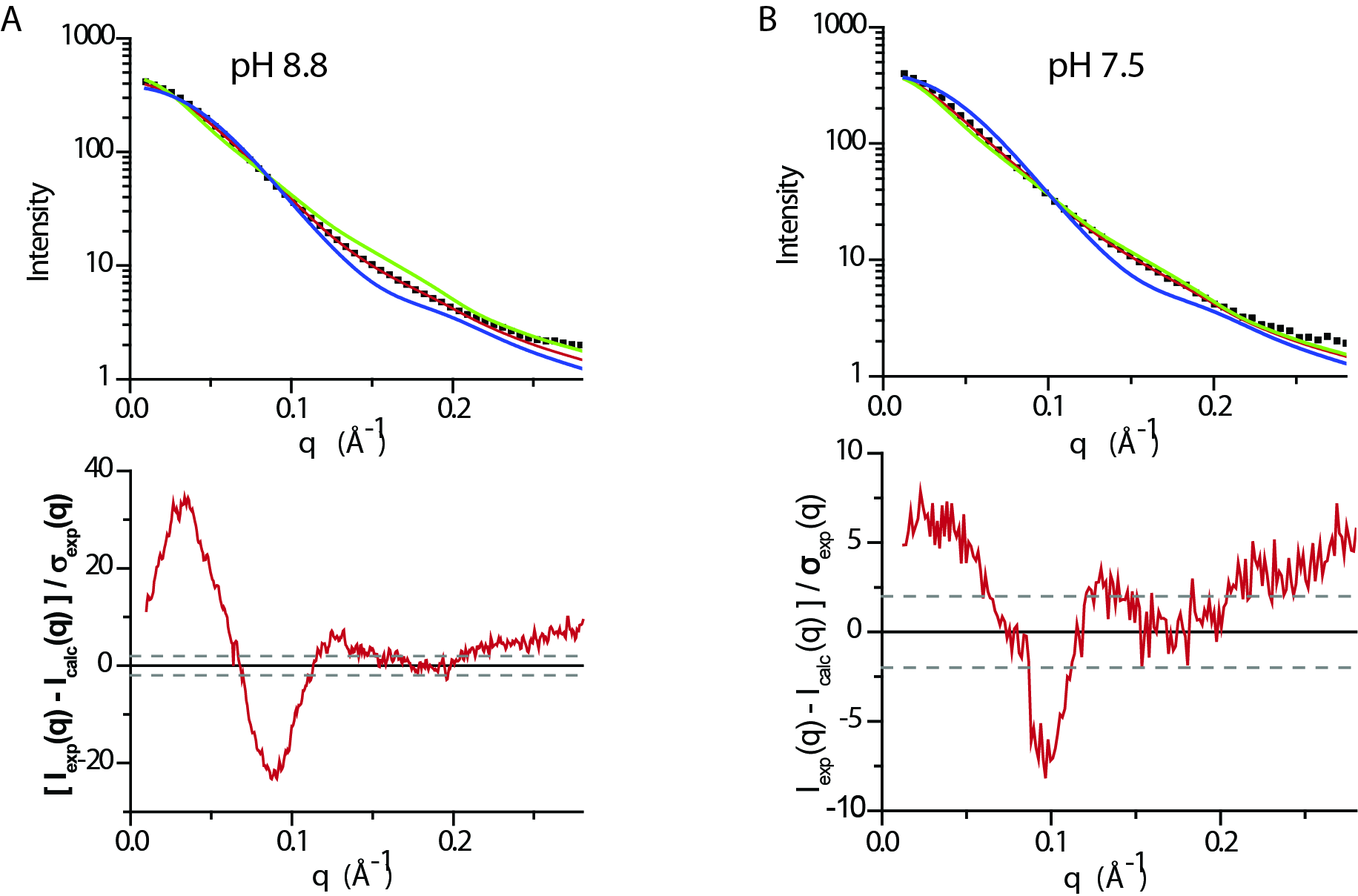

Supplement: Figure S2 — Calculated scattering intensity of pre- and post- fusion crystal structures of protomers and best fit of their linear combinations vs experimental data at pH 8.8 and pH 7.5. Top row: scattering curves; bottom row: reduced residuals corresponding to the linear combination fit, grey dashed lines indicate +2σ and −2σ. Color code: black dots: experimental data; blue line: calculated intensity of the protomer from the pre-fusion crystal structure; green line: calculated intensity of the protomer from the post-fusion crystal structure; red line: best fit using a linear combination of both pre- and post-fusion protomer calculated curves. (TIF) [file ppat.1002556.s002.tif]

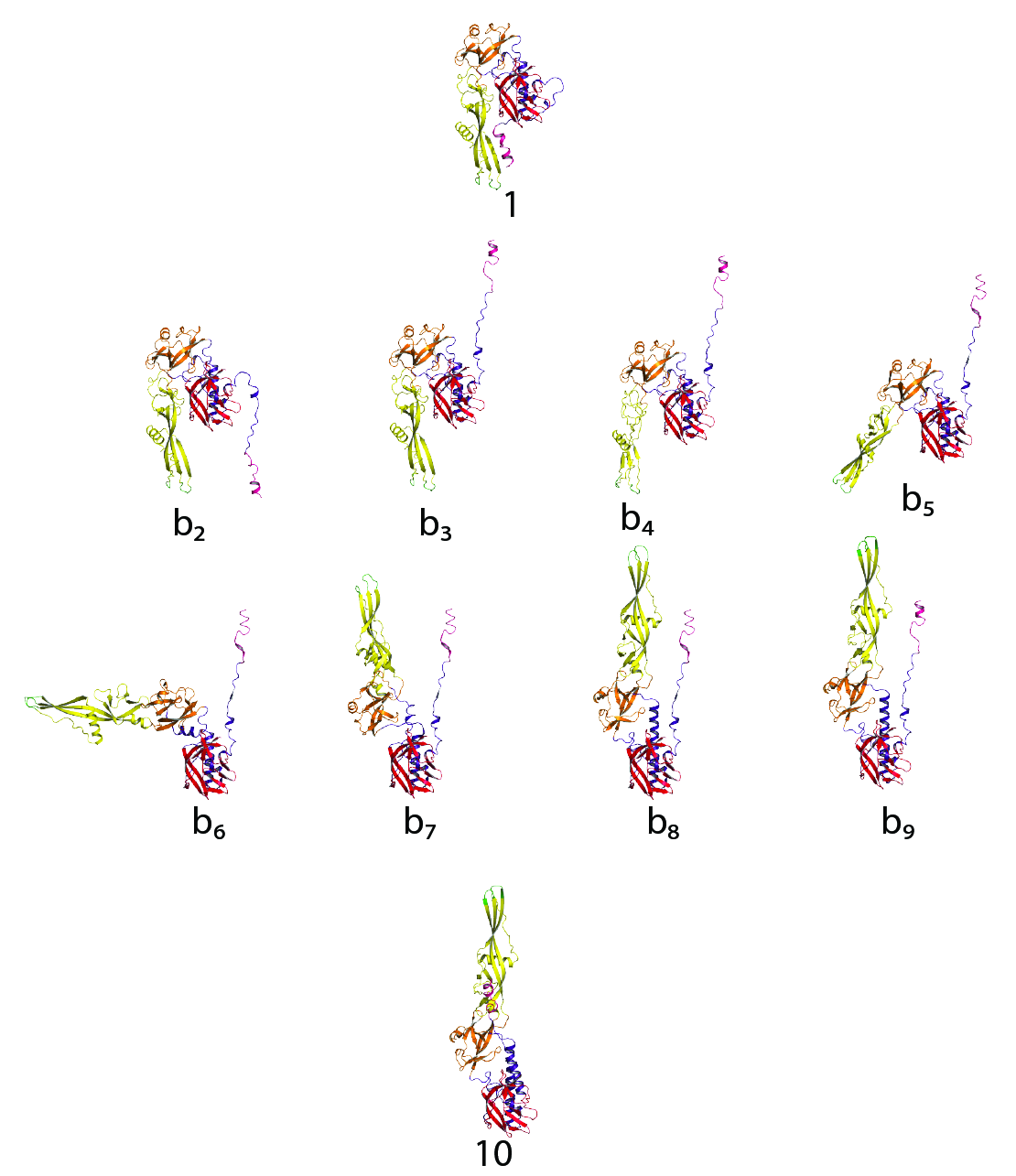

Supplement: Figure S3 — Second set of models of structural intermediates during Gth structural transition in which the C-terminal segment moves first (pathway B). Conformation 1 is the Gth protomer found in the pre-fusion crystalline structure, conformation 10 is the Gth protomer found in the post-fusion crystalline structure. (TIF) [file ppat.1002556.s003.tif]

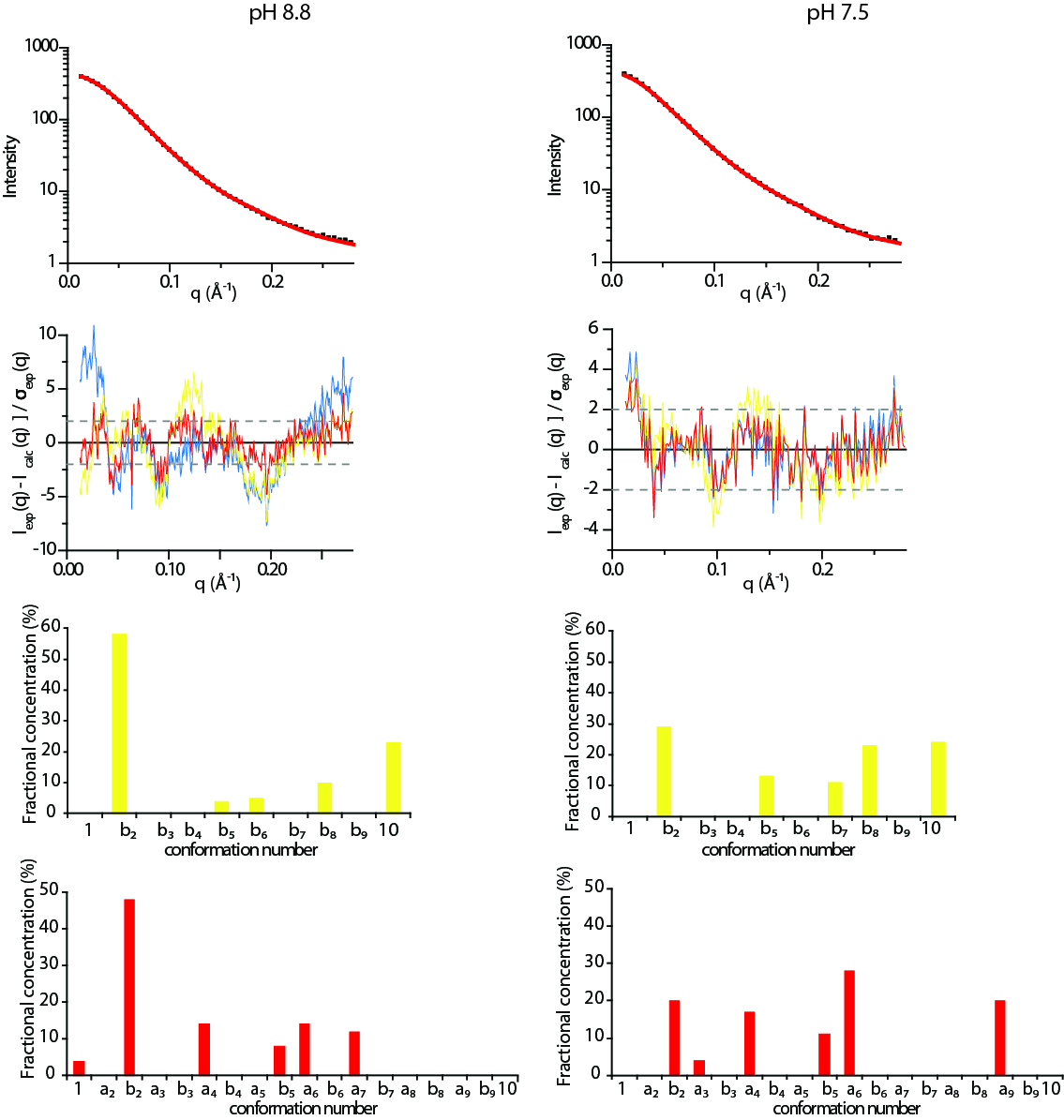

Supplement: Figure S4 — Comparison of fits of experimental data recorded at pH 8.8 and 7.5 using calculated scattering intensities of pathway A (10 models), pathway B (10 models), and combined pathways A and B (18 models). Top row: scattering curves; black dots: experimental data; continuous red line, best fit obtained using all 18 models of combined pathways. Middle row: distribution of reduced residuals corresponding to all three fits. blue: pathway A; yellow: pathway B; red: combined pathways; grey dashed lines indicate +2σ and −2σ. Bottom row: Histograms of the fractional concentrations of each conformation expressed in % of the total population for pathway B (yellow) and combined pathways A and B (blue). (TIF) [file ppat.1002556.s004.tif]

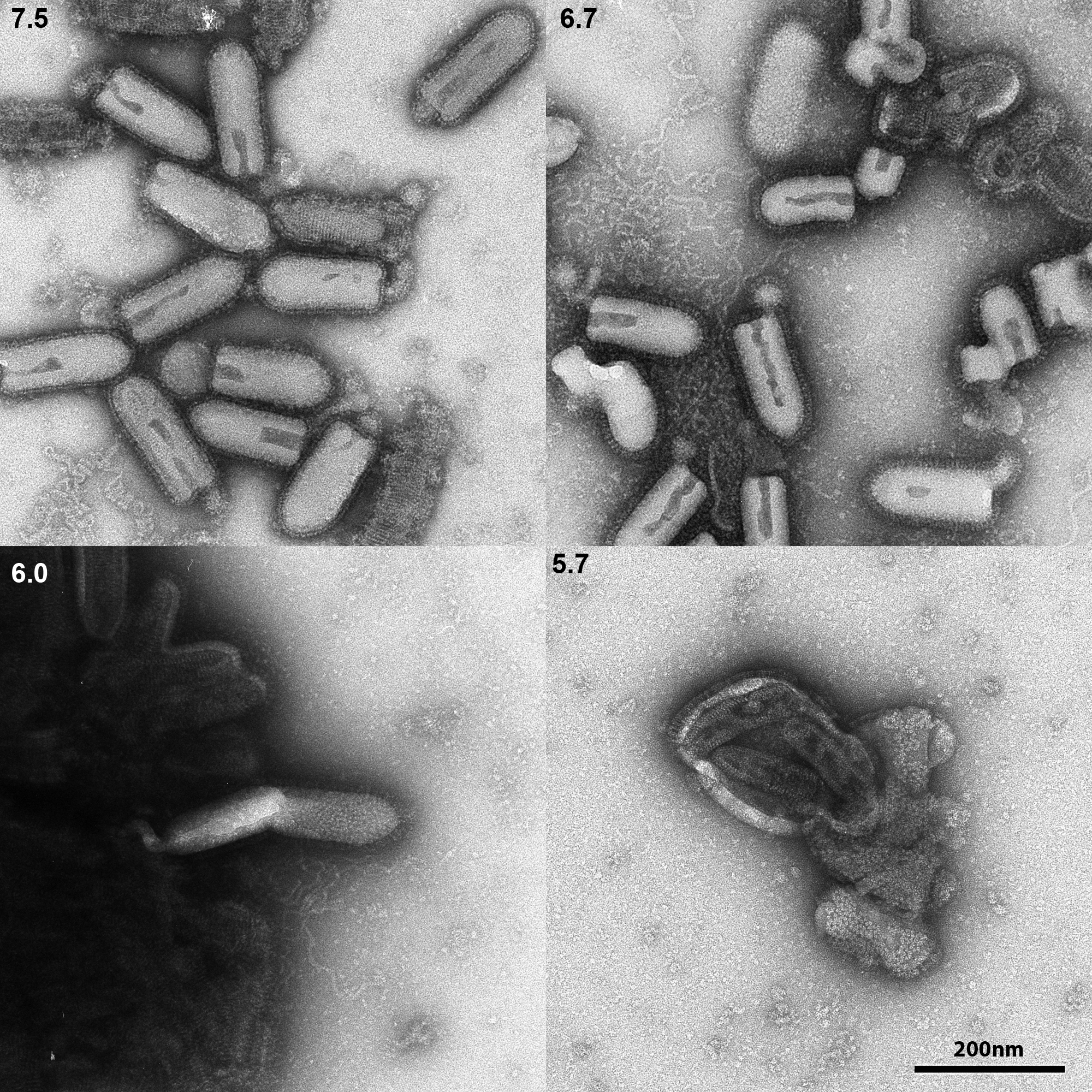

Supplement: Figure S5 — Negatively stained VSV particles at pH 7.5, 6.7, 6.0 and 5.7. All images are at the same magnification (scale bar in the bottom right corner). (TIF) [file ppat.1002556.s005.tif]
