# Supplementary material for: Characterization of Monomeric Intermediates during VSV Glycoprotein Structural Transition
Source: PLoS Pathog. 2012 Feb 23;8(2):e1002556. doi: 10.1371/journal.ppat.1002556 (PMC3285605; doi:10.1371/journal.ppat.1002556)
Supplement: Text S1 — Supporting information for SAXS experiment, data processing and modeling. (DOC) [file ppat.1002556.s006.doc]

**Text S1**

**Small-angle X-ray scattering experiment and data processing.**

X-ray scattering data were collected at Synchrotron SOLEIL (Gif-sur-Yvette, France) on the beamline SWING (experiment at pH 8.8) and on a laboratory instrument (experiments at pH 7.5 and 7.3). At SOLEIL, the wavelength of the X-rays was λ=1.033 Å. Data were recorded using a CCD-based detector (AVIEX) with a sample-detector distance of 1.85 m. Solutions were circulated continuously during data recording through the 1.5 mm diameter quartz capillary using the automatic sample changer (Agilent) at a flow-rate ensuring an irradiation time of less than 1 s [1]. Under these conditions, no radiation damage could be detected in preliminary tests. Samples were prepared at 5 concentrations ranging from 1.3 to 8.5 mg/ml in 20 mM Tris-HCl buffer pH 8.8, 20 mM NaCl, 2% glycerol. All measurements were performed at 15 °C. 15 successive frames of 4.8 s each were recorded for protein solutions and buffer alike.

Our laboratory instrument comprises a copper-coated rotating anode generator Microstar (wavelength of Cu-Kα ray λ=1.54 Å), multi-layer optics and a three pinhole small-angle apparatus Nanostar with a Vantec 2000 detector from Bruker-AXS. Samples were prepared at 3 protein concentrations ranging from 1.4 to 8.8 mg/ml in 20 mM Tris-HCl buffer pH 7.5, 10 mM NaCl, 2% glycerol. Sample at pH 7.3 (10 mM Tris-HCl buffer pH 7.3, 2% glycerol) was only prepared at 1.1 mg/ml in view of the strong tendency to rosette formation. Scattering intensities are therefore very weak even at moderate q-values, which accounts for the limited quality of recorded data. All measurements were performed at 15 °C.

All data were averaged after normalization to the intensity of the transmitted beam before buffer subtraction using the program package PRIMUS [2]. The forward scattering I(0) and the radius of gyration (R_g_) were evaluated using the Guinier approximation [3] ln[I(q)] = ln[I(0)] – R_g_^2^/3 q^2^ over the q-ranges 0.42 < qR_g_ < 1.38 and 0.48 < qR_g_ < 1.17 at pH 8.8 and 7.5 respectively (0.48 < qR_g_ < 1.13 at pH 7.3), where q=4π sinθ/λ is the momentum transfer, 2θ is the scattering angle. The R_g_ values derived from the two most dilute solutions were practically identical, showing that interparticle interactions were essentially negligible under these conditions. The curves of a dilute, interaction free sample and of the most concentrated solution were spliced after scaling to protein concentration to yield a combined, complete scattering pattern. The distance distribution function p(r) corresponds to the distribution of distances between any pair of volume elements within one particle. It was determined using the indirect Fourier transform method as implemented in the program GNOM [4]. Experimental intensities were put on an absolute scale using water as a reference [5]. From this scaled value of I(0)/c an estimate of the molecular weight can be derived. This requires the knowledge of the partial specific volume of the particle. We have taken into account the presence of two hydrocarbon chains, the sequence of which is known, using values of partial specific volumes of sugars given in [6]. The resulting value for the complete glycoprotein G was $\bar{\text{v}}$ = 0.718 cm^3^/g.

If a solution contains various objects with different shapes, as is the case for a flexible protein exploring a conformational space, the scattering pattern is the average of all scattering patterns of individual species weighted by their fractional concentration. In such a case, the apparent radius of gyration experimentally determined is the root mean square of individual values while the maximal diameter D_max_ is not an average but that of the longest species populated enough to be experimentally detectable. Given the individual scattering patterns, the program Oligomer from the package ATSAS determines the volume fractions [7].

**Modeling**

Plausible monomeric intermediates models with additional C-terminal missing segment (residues 414 to 422 for the pre-fusion state and residues 410 to 422 for the post-fusion state) and sugars were built with coot [8] using the PDB coordinates (2J6J and 2CMZ). Two groups of intermediates were built. The first one (Fig. 4) corresponded to a plausible trajectory in which the fusion domain moves first, the second (Fig. S3) to another plausible trajectory in which the C-terminal segment is displaced first with respect to the core domain.

Scattering patterns of crystal structures or models were calculated using the program Crysol [9]. Crysol has some intrinsic limitations in the determination of the contribution of the hydration layer when the center of mass is outside of the particle, as is the case for several of the models proposed as putative intermediates along the pre- to post-fusion conformation pathways. This problem was overcome using adaptations previously made by M. Petoukhov (EMBL Outstation, Hamburg) to the program in the study of a topoisomerase [10].

**References**

1. David G, Perez J (2009) Combined sampler robot and high-performance liquid chromatography: a fully automated system for biological small-angle X-ray scattering experiments at the Synchrotron SOLEIL SWING beamline. J Appl Cryst 42: 892-900.

2. Konarev PV, Volkov VV, Sokolova AV, Koch MHJ, Svergun DI (2003) PRIMUS - a Windows-PC based system for small-angle scattering data analysis. J Appl Cryst 36: 1277-1282.

3. Guinier A (1939) Diffraction of X-rays of very small angles – application to the study of ultramicroscopic phenomenon. Ann Phys 12: 161–237.

4. Svergun DI (1992) Determination of the Regularization Parameter in Indirect -Transform Methods Using Perceptual Criteria. J Appl Cryst 25: 495-503.

5. Orthaber D, Bergmann A, Glatter O (2000) SAXS experiments on absolute scale with Kratky systems using water as a secondary standard. J Appl Cryst 33: 218 - 225.

6. Lewis MS, Junghans RP (2000) Ultracentrifugal analysis of molecular mass of glycoproteins of unknown or ill-defined carbohydrate composition. Methods Enzymol 321: 136-149.

7. Konarev PV, Petoukhov MV, Volkov VV, Svergun DI (2006) ATSAS 2.1, a program package for small-angle scattering data analysis. J Appl Cryst 39: 277-286.

8. Emsley P, Cowtan K (2004) Coot: model-building tools for molecular graphics. Acta Crystallogr D Biol Crystallogr 60: 2126-2132.

9. Svergun DI, Barberato C, Koch MH (1995) CRYSOL - a program to evaluate X-ray solution scattering of biological macromolecules from atomic coordinates. J Appl Cryst 28: 768-773.

10. Graille M, Cladiere L, Durand D, Lecointe F, Gadelle D, et al. (2008) Crystal structure of an intact type II DNA topoisomerase: insights into DNA transfer mechanisms. Structure 16: 360-370.
